# Clinical efficacy of ultrasound doppler-guided hemorrhoidal artery ligation combined with procedure for prolapse and hemorrhoids in treatment of severe hemorrhoids

**DOI:** 10.1097/MD.0000000000036189

**Published:** 2023-11-24

**Authors:** Feng He, Xiao Zhang, Dan Lu, Ziming Wang

**Affiliations:** a Department of Colorectal Surgery, The First Affiliated Hospital of Guizhou University of Traditional Chinese Medicine, Guizhou 550002, China.

**Keywords:** doppler-guided hemorrhoidal artery ligation, hemorrhoids, procedure for prolapse and hemorrhoids, visual analogue scale, Wexner anal incontinence score

## Abstract

Hemorrhoids are a prevalent anorectal condition that affects a wide range of adult populations. The severity of this condition was graded using a validated hemorrhoidal grading system, specifically focusing on grade III and IV cases. This retrospective study aimed to compare the clinical efficacy of a standard Procedure for Prolapse and Hemorrhoids (PPH) with a combined Doppler-guided Hemorrhoidal Artery Ligation (DG-HAL) and a PPH approach in patients with severe hemorrhoids. Conducted from May 2021 to January 2023, the study included patients aged 18–65 with confirmed diagnosis of Grade III or Grade IV hemorrhoids. Patients with a history of anorectal surgery and significant comorbidities were excluded. The control group underwent standard PPH, whereas the observation group received DG-HAL followed by PPH. Clinical outcomes were measured using variables such as the operative duration, intraoperative blood loss, postoperative wound healing time, and length of hospital stay. Efficacy was evaluated using a hierarchical scale and a visual analog scale (VAS) for postoperative pain. The complication rates were also assessed. baseline characteristics were homogeneous between the 2 groups. The observation group demonstrated significantly faster postoperative wound healing and shorter hospital stay (*P* < .01). The overall therapeutic efficacy in the observation group was 90.0%, which was higher than that of the control group (75.0%; *P* = .025). The VAS pain scores were also significantly lower in the observation group (*P* = .002). A marked decrease in complication rates was observed in the observation group (3.3%) compared with that in the control group (17.9%) (*P* < .05). The combined DG-HAL and PPH surgical approach exhibited superior clinical efficacy in treating severe hemorrhoids. This technique offers high effectiveness, reduced postoperative VAS pain scores, and lower complication rates. The long-term efficacy requires further observation.

## 1. Introduction

The prevalence of hemorrhoidal diseases has significantly increased in recent years. This uptick is primarily linked to changes in contemporary society, including sedentary lifestyles, increased stress levels, and unhealthy dietary practices such as low-fiber diets.^[1,2]^ Additionally, advances in diagnostic methodologies have facilitated more accurate and higher reported rates of this condition.^[3]^ The etiological factors responsible for hemorrhoids are complex and often interrelated. Anatomically, variations in the vasculature and muscular support of the anorectal region can predispose individuals to developing hemorrhoids.^[4]^ Furthermore, microbial flora imbalances can contribute to localized inflammation, thereby exacerbating the condition.^[5,6]^ Dietary habits, particularly a low intake of fiber and high consumption of processed foods, can lead to constipation, which is a significant risk factor for hemorrhoidal development. Genetic predispositions also play a role, suggesting that familial history can be indicative of one likelihood to develop this ailment.^[7,8]^ Severe cases of hemorrhoidal disease present a major clinical concern, often characterized by excruciating pain, rectal bleeding, and prolapsed hemorrhoidal tissue, which can severely impede normal bowel function and significantly deteriorate the quality of life of the affected individuals.^[9–11]^

Traditional surgical treatments for hemorrhoids often involve invasive procedures, with significant postoperative complications. Doppler-guided Hemorrhoidal Artery Ligation (DG-HAL) has emerged as a minimally invasive technique that capitalizes on the principles of targeted hemorrhoidal artery ligation. This results in gradual regression of the hemorrhoidal plexus, offering benefits of reduced trauma, a high safety profile, and minimal invasiveness.^[12,13]^ However, DG-HAL is primarily recommended for grade II to III hemorrhoids, characterized mainly by bleeding. Stapled Hemorrhoidopexy, also known as prolapse and hemorrhoids (PPH), employs a mechanical stapling device to excise a circumferential strip of mucosa, effectively reducing prolapse. It has been broadly applied to hemorrhoids across various stages4. Nevertheless, recent studies have pointed out suboptimal long-term outcomes following PPH, predominantly due to incomplete interruption of hemorrhoidal arterial flow, leading to a higher risk of recurrence.^[14]^

The combination of DG-HAL with PPH offers a compelling synergistic approach. Both techniques are minimally invasive, with high safety profiles, and crucially, each complements the limitations of the other.^[15]^ Moreover, this combination preserves the functionality of anal cushions, which is of paramount importance for normal anorectal function. Given the limitations of the current surgical options, there is an urgent need to evaluate the clinical efficacy of this combined technique for treating severe hemorrhoids. This study aimed to critically examine the clinical efficacy of ultrasound-guided hemorrhoidal artery ligation in conjunction with Stapled Hemorrhoidopexy for the management of severe hemorrhoidal disease.

## 2. Materials and methods

### 2.1. Study design

This retrospective study was conducted between May 2021 and January 2023 at the First Affiliated Hospital of Guizhou University of Traditional Chinese Medicine. This study aimed to provide an in-depth analysis of patients with severe hemorrhoids, focusing specifically on Grade III and Grade IV cases, as defined by the validated hemorrhoidal grading system.

The inclusion criteria were as follows: patients aged between 18 and 65 years. Confirmed diagnosis of grade III or IV hemorrhoids. For Grade III hemorrhoids, the hemorrhoidal cushions must have required manual reduction following prolapse, whereas for Grade IV, the prolapsed hemorrhoidal cushions were irreducible or would prolapse again after reduction. Presence of 3 or more hemorrhoidal cushions.

The exclusion criteria were as follows: patients with a previous history of hemorrhoidectomy or other anorectal surgical interventions. Patients with a previous History of hemorrhoidectomy or other anorectal surgical interventions. The coexistence of other anorectal diseases such as tuberculosis, rectal cancer, or Crohn disease. Patients with significant comorbid conditions leading to organ dysfunction.

The study protocol was reviewed and approved by the Ethics Committee of the First Affiliated Hospital of Guizhou University of Traditional Chinese Medicine. All participants were informed of the study objectives, potential risks, and privacy safeguards, and written informed consent was obtained prior to the data collection.

### 2.2. Surgical procedure

Control Group: Patients in the control group underwent standard PPH. Preoperatively, patients were subjected to fasting and enemas for bowel cleansing. Subarachnoid block anesthesia was administered while the patients were in the lateral decubitus position. Dilatation of the anal canal was performed using a specialized anal dilator (registered under the National Medical Products Administration). The hemorrhoidal tissue was repositioned, and purse-string sutures were placed above the dentate line at approximately 9 and 3 o’clock positions using 2-0 absorbable sutures. An anastomotic stapler was then inserted and positioned. After confirming adequate tissue depth (4.0 cm, the stapler was fired and kept closed for 30 seconds. After the procedure, if pulsatile bleeding was observed, figure-8 sutures were applied using 3-0 suture material; otherwise, a hemostatic gauze was inserted. Postoperative care included bed rest, a 12-hour fasting period, and antibiotic prophylaxis to prevent infection.

Observation Group: Procedure for DG-HAL Followed by PPH. The observation group underwent the same preoperative preparation and anesthesia as the control group. Initially, a Doppler ultrasound probe (ELCAT GmbH, Handydop-pro, Germany) was used to identify hemorrhoidal arteries with strong blood flow signals. The targeted arteries were then ligated using figure-8 sutures with 3-0 absorbable suture material. Doppler ultrasound was again employed post-ligation to verify reduced blood flow. The procedure was subsequently followed by standard PPH as performed in the control group.

### 2.3. Evaluation criteria and outcome measures

In this study, we employed a series of well-defined metrics to compare the clinical outcomes between the 2 treatment groups undergoing distinct surgical procedures for hemorrhoids. Key performance indicators included the operative duration, intraoperative blood loss, postoperative wound healing time, and length of hospital stay. The treatment outcomes were graded using a hierarchical efficacy scale and categorized into Complete Remission, Significant Improvement, Moderate Improvement, and No Change. The efficacy rate was calculated as (number of complete remission + number of significant improvements + number of moderate improvements)/ Total Number of Cases × 100%. Furthermore, pain intensity levels at baseline, postoperative day 7, and day 14 were gauged using the visual analog scale (VAS), with scores ranging from 0 to 10, where 0 denotes no pain and 10 signifies intolerable pain. The Wexner Anal Incontinence Score was used to assess the frequency of incontinence across the 5 types, scored from 0 to 20, where 0 is normal and 20 denotes complete incontinence. We also conducted a comparative analysis of the incidence rates of postoperative complications at the 3-month mark, including bleeding, perianal edema, urinary retention, anal distention, and perianal abscess formation.

### 2.4. Statistical analysis

Statistical evaluations were performed using IBM SPSS Statistics version 27.0. Prior to any inferential analysis, the collected data were examined for a normal distribution. The dataset was then divided into continuous and nominal variables. For continuous variables conforming to a normal distribution, intergroup disparities were scrutinized through the application of independent sample *t* tests. The outcomes were subsequently articulated as arithmetic means along with their corresponding standard deviations (expressed as mean ± SD). Conversely, nominal variables were represented as frequency counts and proportional percentages, and their associations or independence was assessed using chi-square (χ^2^) tests. All statistical tests were executed as 2-tailed hypotheses, with the significance level (alpha) set at 0.05. This alpha level was selected in compliance with well-established scientific criteria, thus optimizing the trade-off between Type I and Type II errors.

## 3. Results

### 3.1. Baseline clinical attributes of study participants

In our study, the baseline characteristics of the observation and control groups were closely matched, indicating the comparative validity of the 2 cohorts. The variables assessed included sex distribution, age, average duration of illness, hemorrhoidal staging, and hemorrhoidal count. The p-values obtained from the *t* test or chi-square test for these variables were all >0.05, suggesting that the differences were not statistically significant. This validated the homogeneity of the 2 groups, rendering them suitable for comparative analysis (Table 1).

**Table 1:** Comparative baseline characteristics of patients in the observation and control groups.

Group	Gender (n)	Age (yr)	Avg. duration of illness (yr)	Hemorrhoidal staging (n)	Hemorrhoidal count
Control (n = 56)	M: 28/ F: 28	43.74 ± 9.12	8.05 ± 3.53	Stage III: 26/ Stage IV: 30	1.71 ± 0.49
Observation (n = 60)	M: 27/ F: 33	44.26 ± 9.42	8.18 ± 3.59	Stage III: 23/ Stage IV: 37	1.69 ± 0.45
t/χ² value	3.08	2.08	1.76	3.47	1.88
*P* value	.068	.059	.077	.062	.065

F = female, M = male.

### 3.2. Comparative assessment of surgical outcomes

The evaluation of surgical outcomes included variables, such as surgical time, intraoperative blood loss, postoperative wound healing time, and length of hospital stay. While the first 2 parameters showed no statistically significant differences between the observation and control groups (*P* > .05), the latter 2 parameters were noticeably different. Specifically, the observation group demonstrated significantly faster postoperative wound healing and shorter hospital stay (*P* < .01). This may suggest that the treatment protocol employed in the observation group was more efficacious in accelerating recovery, thereby reducing the overall burden on healthcare resources (Table 2).

**Table 2:** Comparative analysis of surgical parameters between observation and control groups.

Group	Surgical time (min)	Intraoperative blood loss (mL)	Postoperative wound healing time (d)	Length of hospital stay (d)
Control (n = 56)	40.11 ± 11.25	62.12 ± 20.41	22.68 ± 3.78	11.61 ± 4.19
Observation (n = 60)	42.46 ± 10.79	66.21 ± 19.25	16.88 ± 4.09	6.83 ± 3.55
t value	0.75	0.95	6.92	5.63
*P* value	8.65	9.67	.003	.004

### 3.3. One-month postoperative therapeutic efficacy

The therapeutic efficacy assessed 1 month postoperatively was significantly higher in the observation group than in the control group. The overall efficacy rate in the observation group was 90.0%, which was markedly higher than the 75.0% reported in the control group (*P* = .025). Notably, the observation group exhibited a greater number of complete recoveries and significant effects, emphasizing the effectiveness of the intervention (Table 3).

**Table 3:** Comparative analysis of 1-mo postoperative therapeutic outcomes between observation and control groups.

Group	Recovery (n, %)	Significant effect (n, %)	Effective (n, %)	Ineffective (n, %)	Overall efficacy rate (%)
Control (n = 56)	36 (64.3)	3 (5.4)	3 (5.4)	14 (25.0)	42 (75.0)
Observation (n = 60)	44 (73.3)	6 (10.0)	4 (6.7)	6 (10.0)	54 (90.0)
χ^2^ value					5.04
*P* value					.025

### 3.4. Evaluation of pain and anal incontinence through vas and Wexner scores

Pain and anal incontinence were systematically evaluated using the visual analog scale (VAS) and Wexner scoring systems at different time points. Statistical data showed that the observation group outperformed the control group in terms of reducing anal incontinence, as evidenced by a significantly lower Wexner score at the 3-month postoperative assessment (*P* = .001). Furthermore, the 14-day postoperative VAS scores were substantially reduced in the observation group compared to those in the control group, indicating improved pain management (*P* = .002, Table 4).

**Table 4:** Comparative analysis of VAS and Wexner scores for anal incontinence in observation and control groups at preoperative and postoperative intervals.

Group	Preoperative VAS score (x ± s)	7d postoperative VAS score (x ± s)	14d postoperative VAS score (x ± s)	3-mo Postoperative Wexner score (x ± s)
Control (n = 56)	0.66 ± 0.20	2.33 ± 0.65	3.50 ± 0.89	5.73 ± 0.42
Observation (n = 60)	0.69 ± 0.18	2.37 ± 0.68	2.03 ± 0.65	2.37 ± 0.55
t-value	0.22	0.27	7.95	28.50
*P* value	14.700	14.150	.002	.001

VAS = visual analogue scale.

### 3.5. Postoperative complications

postoperative complications were monitored in both groups. The data revealed a statistically significant reduction in the total incidence of complications in the observation group (3.3%) compared to the control group (17.9%) (*P* < .05). Specifically, the occurrences of perianal edema, urinary retention, and anal prolapse were markedly lower in the observation group (Table 5).

**Table 5:** Comparative analysis of postoperative complications between observation and control groups.

Group	Perianal edema (n)	Urinary retention (n)	Anal prolapse (n)	Total incidence of complications (%)
Control (n = 56)	3	5	2	17.9
Observation (n = 60)	1	1	0	3.3
*P* value	<.05	<.05	<.05	<.05

## 4. Discussion

In the ever-evolving discourse on the etiology of hemorrhoids, 2 prevailing theories currently dominate literature. The first posits that hemorrhoids arise from varicosities of the venous plexus located beneath the mucous membrane in the lower rectum and anorectal skin, commonly referred to as the varicose theory.^[16,17]^ However, more recent insights, particularly from Western medicine, point toward the concept of “anal cushion prolapse,” or anal cushion descent theory, initially posited by THOMSON.^[18]^ From this perspective, hemorrhoids are considered a pathologically descended form of normal anatomical structures, known as vascular cushions located 1.5 cm above the dentate line. Based on the anal cushion descent theory, the procedure for PPH is designed to reposition and fix the descended cushions to their anatomical position, particularly in patients with Grade III and IV internal hemorrhoids.^[19–22]^ Another minimally invasive technology, DG-HAL, has shown particular utility in treating Grade III and IV internal and mixed hemorrhoids. However, studies have indicated that PPH may result in long-term adverse effects, including high recurrence rates, rectal perforation, and pelvic abscesses. DG-HAL, which ligates hemorrhoidal arteries, offers limited benefit in the fixation of anal cushions and is generally employed for patients with hemorrhoids characterized by severe bleeding.^[23–26]^

Our study aimed to evaluate the clinical efficacy of combining DG-HAL with PPH versus PPH alone for the treatment of severe hemorrhoids. The results demonstrated no significant difference in the duration of surgery and intraoperative bleeding between the 2 groups (*P* > .05), but revealed a notably shorter healing time and hospital stay for the combined group (*P* < .05). The efficacy of DG-HAL in ligating hemorrhoidal arteries and inducing tissue fibrosis aids the reduction of hemorrhoids and prevents future prolapse. In our study, the observation group initially underwent DG-HAL with absorbable No.3 sutures. These sutures offer a longer degradation time in the body than the time required for hemorrhoidal tissue to slough off, averting the risk of wound dehiscence. In contrast, PPH alone, employing purse-string sutures and stapling, could potentially result in wound dehiscence and infection, prolonging healing and hospital stay.

The Wexner incontinence score, assessment of anal function through multiple parameters, including incontinence to solid, liquid, and gas, use of pads, and lifestyle changes, indicated better results for the observation group at 1-month post-op and significantly lower scores at 3 months (*P* < .05), suggesting superior short-term outcomes with the combined approach. Our study also revealed no significant difference in pre-op and 7-day post-op VAS pain scores, but a significantly lower VAS score at 14 days post-op in the combined approach group (*P* < .05), indicating effective pain relief. Moreover, PPH alone has been criticized for its higher rates of long-term recurrence and lack of a curative effect due to factors such as high anastomotic levels, ineffective ligation of hemorrhoidal arteries, and inability to deal with enlarged anal cushions.^[27]^ Our study suggests that the combined approach can compensate for these deficiencies. For instance, 80% of patients undergoing PPH alone still had detectable hemorrhoidal arterial flow 3 months post-op, whereas the combined approach could eliminate this drawback through precise ligation by DG-HAL.^[28]^ This implies a potentially lower rate of recurrence and postoperative pain, although long-term follow-up is required to obtain conclusive evidence.

In the realm of proctology, hemorrhoids present an enduring challenge with a dire need for refining therapeutic techniques that promote optimal outcomes and diminished morbidity. This study strived to innovate by amalgamating 2 surgical techniques, Doppler-guided Hemorrhoidal Artery Ligation (DG-HAL) and Procedure for Prolapse and Hemorrhoids (PPH), to address severe hemorrhoids.

The novelty of our research primarily resides in the integration of these procedures. While PPH is acknowledged for its rectal mucosal resection, which diminishes prolapsed tissue, it doesn’t explicitly target the hemorrhoidal arteries. Contrarily, DG-HAL strength lies in its ability to pinpoint and ligate these arteries, considerably slashing the blood supply to the hemorrhoids. Consequently, coupling these techniques attempts to harness the advantages of both, aiming for enhanced efficacy and a more favorable side-effect profile. Our results suggest that this combination could be revolutionary in the field. Not only was there a significant reduction in the postoperative healing time and length of hospital stay, but also a higher therapeutic efficacy rate was observed in the combined method group. Moreover, a marked decrease in postoperative complications and improved pain management underscores the prospective benefits of this approach. While DG-HAL and PPH have been independently associated with reduced postoperative pain and complications in past research, the synergistic benefits witnessed in this study have not been documented before, introducing a potentially transformative dimension to hemorrhoid treatment. Furthermore, the repercussions of this study stretch beyond mere clinical outcomes. Reducing the length of hospital stay and complications has economic implications, potentially leading to decreased healthcare costs and resource allocation. This presents the prospect of making treatment more accessible and affordable, a crucial consideration in today resource-constrained healthcare environments. Lastly, our findings also pave the way for future research, delving deeper into the long-term effects, optimizing the technique, and making it accessible to a broader demographic.

While the present study offers valuable insights into the combined efficacy of DG-HAL and PPH surgeries for treating severe hemorrhoids, it is not without its limitations. First, the follow-up period for assessing long-term recurrence rates was insufficient, thereby limiting the understanding of the sustained therapeutic effects of the surgical approaches. Second, the study did not establish a uniform standard for PPH suture height, which is a critical factor affecting postoperative outcomes. Furthermore, the sample size was relatively small, which reduces the generalizability of the findings. Lastly, the study was conducted in a single institution, which may have introduced bias and limited the external validity of the results. These limitations necessitate further multicenter large-scale studies to validate these findings.

## 5. Conclusions

In summary, the combined DG-HAL and PPH surgical approach demonstrated promising clinical efficacy in treating severe hemorrhoids. The method is highly effective, significantly lowers postoperative VAS pain scores, and maintains a high safety profile with a low incidence of complications. However, the long-term effectiveness of this treatment strategy requires further investigation.

## Acknowledgments

We are grateful for the technical assistance provided by the clinical research staff and students of our laboratory. We appreciate the patients’ participation and informed consent for this study.

## Author contributions

**Data curation:** Xiao Zhang.

**Formal analysis:** Feng He, Dan Lu.

**Investigation:** Xiao Zhang.

**Methodology:** Xiao Zhang.

**Resources:** Feng He, Dan Lu.

**Software:** Dan Lu.

**Supervision:** Ziming Wang.

**Visualization:** Ziming Wang.

**Writing – original draft:** Feng He.

**Writing – review & editing:** Ziming Wang.
